# Daily Jottings in the Gold Book of a Dentist

**Published:** 1853-04

**Authors:** E. Townsend


### ARTICLE VII.

*Daily Jottings in the Gold Book of a Dentist.* By E. Townsend, D. D. S., M. D.

### No. II.

Mr. S. W. C. a young gentleman aged 22 years, applied for relief. Complained of very great soreness in the second inferior molaris, approximal surface. A young man, who had been in the practice of dental surgery a short time, had attempted to destroy the nerves and had failed. He had once filled the cavity in the tooth, but, the pain becoming unendurable, was obliged to remove the filling. He had filled the crown only. Upon examination, it was found that with a timidity common to young operators, he had not allowed himself sufficient room to do his work thoroughly. After making a free space between the teeth with my cutters, and file, thereby thickening and strenghening the margin of the cavity, I proceeded to probe the fangs, in which I found the exposed, but not insensible membranes, in a very unpleasant and fetid state. After picking out as much as was possible at one sitting, a pledget of cotton saturated with chloride of zinc was introduced and carefully covered with wax. The following day the remaining portions of the membranes sloughed and came away to the end of the fangs, leaving the cavities perfectly dry and clean, and almost free from odor. The soreness in the peridental membrane was somewhat diminished, though still too great to permit the closing of the mouth with comfort. I next made a deep incision in the gum opposite, or as nearly so as possible to the apices of the fangs, and introduced a piece of cotton to act as an issue. This was changed every other day, and the soreness rapidly diminished under this treatment. After two weeks had elapsed from the first call, I determined to fill the fangs, and doing so, passed the gold as far down to the apices as possible, using No. 30, cut into narrow strips. When the fangs were filled to the floor of the main cavity, and the gold well burnished, the patient was dismissed with an appointment four days in advance. When next he called, I found the irritation had not yet subsided, and was induced to believe it was kept up by the occlusion of the jaws, as the molar which I was endeavoring to save, met its antagonist fairly and flatly. Having a tooth to fill in the end of the crown on the opposite side of the mouth, I decided to fill this with gold in such way as to prevent the mouth from closing, which was effected by raising the gold above the surface of the crown, and thus entirely preventing the affected tooth from touching its antagonist. In ten days I had the satisfaction of finding the soreness entirely removed, and also of filling the crown cavity in the firmest manner: I then trimmed away the filling which had been used to prevent close occlusion of the jaws, and have since had the satisfaction to know there has been no soreness or other uncomfortable feeling in the tooth—it is as useful as any other, and the gum and all its surroundings in perfectly healthy condition.

Mr. J. consulted me on behalf of his wife, who had been suffering from the filling of a tooth. She was seven months advanced in gestation, had felt some pain in the right superior molar, and her dental adviser, after examining her mouth, informed her he would not put in any thing to save her the pain of the extirpation of the nerve; if he took the nerve out, or in other words destroyed it, he should crush it in with an instrument, but he thought he could fill over it, and around it, so that it would do very well. Though it might give her pain for a while, it would pass off after an abscess should form, and he had no doubt the tooth would do in that way as well as any other. The lady being very sensitive and nervous, said she could not then submit to have the nerve crushed in, or cut, and must therefore have it filled over the exposed nerve. This was accordingly done, and, as far as I could judge from her representation, without any particular care to prevent a somewhat too forcible pressure on the inflamed vessels. The pain increased in violence from the time the tooth was filled, and when her husband called upon me, in reply to my remark, "They should consult with the dentist who had filled it, as he was a man who held high rank," said he had done so and the answer was, "She must bear it until an abscess forms, and then it will get well of itself." I called to see the lady at her house, as she was not able to come to me at once, owing to her suffering and loss of rest. Upon questioning her closely I found the irritation had so fully reported itself in distant organs that she had felt uterine pains, which she feared might produce some trouble, and I had also some fear that premature parturition might be the consequence of protracted suffering. Examining the tooth I found it sore to the slightest pressure, and an evident engorgement of the blood-vessels in the neighborhood. After removing the filling, which was tolerably solid, I used a broad-edged cutter and removed the thin carious portion of bone over the nerve canal, with one quick cut, and a profuse flow of bloody serum, and almost immediate relief from pain was the result.

The tooth was left open to free itself until the following day, when the pain having entirely subsided, and the soreness of the external membranes very much lessened, a probe was passed into the fangs to ascertain the exact state of feeling of the nerve. This was found to have some vitality about half way up the palatal fang, without removing any even of the outer dead portions of the pulp, arsenious acid and kreosote were placed in the cavity, and carefully sealed over with wax. The reasons for leaving the portions of the pulp already dead, untouched, are two; in the first place, removing them until you reach the living portion, excites some flow of blood which renders it more difficult to make the acid act as perfectly as we wish, and second, this dead portion is a good conductor of the arsenic to the living, without the danger of producing pain by pressure in the application. After leaving the preparation in the cavity twenty-four hours, removed it, and found the hair-like probe could be passed to the apex of the fang without pain, and therefore extirpated the pulp from the palatal root entire, the buccal roots being very small were removed with more labor and difficulty. The external cavity was then filled with cotton, loosely, so as to keep out foreign matter, but not prevent the free flow of serum from the fangs into the cotton. Three days after, finding the tooth free from soreness, pain or other unfavorable symptoms, and that the nerve cavities were dry and clean, I filled the fangs, and on the following day the crown, and have since been gratified to know that the tooth has been in all respects as useful and comfortable as before it was attacked by caries.

The advantage derived from filling the first permanent molars has been very clearly proven to me in the teeth of Miss C., even when, as in her case, from early and incipient caries they are very tender, and are almost certain to be entirely lost by the eighth or ninth year. At the time the two inferior and two superior molars were filled she was seven years old, the two latter teeth just through the gum, the bony structure was soft and exceedingly tender. They were filled with great difficulty at the time, as she was very timid, and it was utterly impossible entirely to remove the softened portions; indeed it had so little density that it was difficult to decide the exact line between the sound and carious portions. The fillings, which were of gold, were however placed in the cavities dry, and as solid as possible under the circumstances, and an injunction strongly urged that the parents would attend to re-filling them from her fourteenth to sixteenth year. I expressed a hope to her and the parents, by that time the bony structure would have acquired solidity, and it would then be possible to re-fill them thoroughly and without pain. The result has proved my anticipations correct, it has also proved that they would in her case have been a great loss as her teeth are not crowded; indeed, there is a space between all of them, and the loss of these teeth at that age would inevitably have brought the bicuspids through the gum at a distance from each other almost sufficient for a tooth between them. She is now seventeen, and the teeth have been firmly re-filled, and without pain, the bone had become hard, and the portions once soft being perfectly protected, even by the fillings done under such disadvantages, had acquired such hardness that little scraping was necessary to prepare them for a more solid and durable filling. They are likely to last as well as any of her teeth, even those which have not been attacked by caries.

The dependance of one branch of the healing art upon the others is very often evidenced, and should lead every practicing dentist to study well all the collateral branches or sciences that can in any way, directly or otherwise, bear upon his own. As he cultivates the general study of medicine, and is known to possess other knowledge besides the mechanical manipulations of his art, he will be recognized as a man of general science, and be called into consultation with the general surgeon and physician, for that special knowledge which he is expected to have. The occulist can often be very much assisted by the dentist in his efforts to relieve the diseases of the eye, particularly those which seem to arise from nervous irritation; sometimes this irritation reports itself by an intolerance of light, as in a case which has recently fallen under my own notice, and in which the occulist desired consultation. The patient, a young gentleman of 19½ or 20, has been unable for the past year to open his eyes upon the day light, and even in the evening going out with a thick shade over his eyes, a kind of a close fitting pad. He had never had any pain or uneasiness in his teeth, and prior to coming to the city for advice, his father wrote to the gentleman under whose care he has placed himself, whose reply was, "first have his teeth carefully examined," to which the attending physician replied, "no need to look at his teeth, they have nothing to do with it." An examination of his mouth proved to me that freedom from caries his teeth were perfect, there was some tartar about the necks, and the gums were a little inflamed from this cause, but not sufficiently to give me any reason to hope it could lead to a solution of the mystery. An appointment was made however to scale these teeth, and make further examinations. In conversation at this sitting, I learned that the commencement of the intolerance of light had been coincident with the eruption of the wisdom teeth, and that it had been increasing in intensity as the teeth advanced. On sounding the teeth very carefully, and sounding all the other molar teeth, and then comparing the differences of sound, I inferred that the fangs of these wisdom teeth were yet in a state of formation, and, as his development had evidently outdone the capability of his system of formation in a perfect way, and a general feebleness of tone was thereby induced. I believed it to be barely possible the irritation was caused by the effort of the formative process in these teeth, and that it was acting on some of the associate branches of the opthalmic nerve: one word as to my associate manner of sounding these teeth, so that the truest resonance should be carried to my ear. I place one end of a bar of steel upon the tooth and the other in my own mouth, grasping it with my teeth, then strike upon the tooth with a sound, and I have it perfectly. I determined to extract the two superior wisdom teeth, and when I had done so, found they each had four distinct fangs, well defined, wanting about a line of their finish, and an appearance of greater than healthy vascularity in the unossified portion of the apices. I congratulated the son and the father, and told them I hoped to hear he was better before many days. One week afterwards I called to see him, and found him very much improved, in a fortnight he walked out at twilight without his pads, and one day when it was cloudy went out to take the air, but while out the sun broke through, and pained him so much that for some days he was again thrown into the dark room, with increase of the symptoms. Since that time, however, he has been gradually and surely, though slowly, recovering.

A lady called who had been under the care of her attending physician for neuralgia of the side of the head, neck and chest, indeed all of the right side was severely afflicted, and a partially disability of motion of the arm. He had been treating her on the best and most approved principles of the practice of medicine, and after failing to produce any good effect, said to her, "get into my carriage and come with me, there may be something the matter with your teeth." "Oh no, my teeth never hurt me, besides they were all put in order about two months before this attack." "No matter, come." On learning the symptoms, which were told me by herself very clearly, I suspected some tooth had been the whole cause of the trouble. She informed me, in conclusion, she had been more free for a day or two than usual, but very much feared she should be a victim to neuralgia all her life if she even escaped paralysis. A close inspection of her teeth proved to me that the inferior molar in the left side was the exciting cause of the malady, and this brought its history. Her dentist was cruelly kind, the tooth was slightly carious and needed a small filling in the buccal surface; finding it a little tender, he said "oh we will soon fix that," put in some arsenious acid, covered it over, left it three days, then removed it, and immediately filled the tooth, admiring himself for being so painless an operator. The tooth was white, the gum and surrounding parts healthy, but when I pressed it in one direction, a thrill of pain was the consequence, removed the filling, which was very easy, and drilled through a thick plate of dead insensible bone to reach the pulp cavity, when I reached this, a gush of pus of the most fetid character followed the withdrawal of the drill, and in a few minutes entire relief from the pain which had been caused by my pressure. This cavity was enlarged so as to give free vent to the secretion, the pus became more and more healthy, and in two weeks, I filled the fangs, and two days after the external cavity, the neuralgic and paralytic symptoms all having disappeared. She has since had perfect health, and I have had occasion to fill other of her teeth, some of which had the tenderness so common to dentine, but she always said "cut away, dont put any more stuff in to save a temporary pain."

One word here of this practice—it may sometimes be admissible, but it must always be used with much caution, for, if it is put into the tooth under the surface of the enamel, the capillary tubes carry it into the nerve and it destroys the vitality in the worst way. When it is found absolutely indispensable, it should be kept in only a few hours, and even this must be varied to suit the different density of teeth, and after its removal, and the cavity carefully washed with warm water, the whole interior surface should be scraped, taking care not to leave any part untouched until, if possible, you reproduce at least some of the tenderness; by this you have proved that your instrument has reached dentine healthy and unaltered by the acid. In such case you *may* be successful, but even here I would not say be very sanguine, for sometimes months after the tooth will lose color and vitality, and you may be mortified to find you have failed.

### ARTICLE VIII.

*Treatment of Hemorrhage.* By F. L. CRANE, D. D. S., Easton, Pennsylvania.

